# Transcriptome-Wide Analysis of *CXCR5* Deficient Retinal Pigment Epithelial (RPE) Cells Reveals Molecular Signatures of RPE Homeostasis

**DOI:** 10.3390/biomedicines8060147

**Published:** 2020-06-01

**Authors:** Madhu Sudhana Saddala, Anton Lennikov, Anthony Mukwaya, Hu Huang

**Affiliations:** 1 Department of Ophthalmology, University of Missouri, Columbia, MO 65212, USA; saddalam@missouri.edu (M.S.S.); lennikova@missouri.edu (A.L.); 2 Department of Ophthalmology, Institute for Clinical and Experimental Medicine, Faculty of Health Sciences, Linköping University, 58183 Linköping, Sweden; anthonny.mukwaya@liu.se

**Keywords:** age-related macular degeneration, *CXCR5*, EMT, *FoxO*, Mitochondria, RNA-Seq, gene ontology, KEGG, retinal pigment epithelium

## Abstract

Age-related macular degeneration (AMD) is the most common cause of irreversible blindness in the elderly population. In our previous studies, we found that deficiency of *CXCR5* causes AMD-like pathological phenotypes in mice, characterized by abnormalities and dysfunction of the retinal pigment epithelium (RPE) cells. The abnormalities included abnormal cellular shape and impaired barrier function. In the present study, primary RPE cells were derived separately from *CXCR5* knockout (KO) mice and from C57BL6 wild type (WT). The isolated primary cells were cultured for several days, and then total RNA was isolated and used for library preparation, sequencing, and the resultant raw data analyzed. Relative to the WT, a total of 1392 differentially expressed genes (DEG) were identified. Gene ontology analysis showed various biological processes, cellular components, and molecular functions were enriched. Pathway enrichment analysis revealed several pathways, including the *PI3K-Akt* signaling, *mTOR* signaling, *FoxO*, focal adhesion, endocytosis, ubiquitin-mediated proteolysis, *TNFα-NF-kB* Signaling, adipogenesis genes, *p53* signaling, Ras, autophagy, epithelial–mesenchymal transition (EMT), and mitochondrial pathway. This study explores molecular signatures associated with deficiency of *CXCR5* in RPE cells. Many of these signatures are important for homeostasis of this tissue. The identified pathways and genes require further evaluation to better understand the pathophysiology of AMD.

## 1. Introduction

Age-related macular degeneration (AMD) is a progressive degenerative disease affecting mainly the elderly population, and it is known to affect more than 180 million people worldwide [1]. Anti-*VEGF* agents are currently the approved treatment for the "wet" form of AMD. However, no treatments are available for the "dry" form of AMD. Dry AMD accounts for 85~90% of the total reported AMD cases [2,3]. Chemokines are chemotactic cytokines known to control the migration and localization of various cells, such as immune cells and leukocytes. There are about 20–30 chemokine receptors in the mammalian genome, including humans. Chemokine receptors are hypothesized to be essential for the pathogenesis of AMD, as they regulate the migration of immune and inflammatory cells, which contributes to the initiation and development of AMD [4,5]. The *C-X-C motif chemokine receptor 5 (CXCR5)*, a chemokine transmembrane receptor, belongs to the C-X-C chemokine receptor family. *CXCR5* acting via its ligand *CXCL13* plays a crucial role in B cell initiation. Primarily recognized in Burkitt lymphoma, and in other several tumors [6], current studies have shown that the *CXCL13/CXCR5* signaling axis might play an important role in the central nervous system (CNS), for instance, in pain transduction [7].

Recently, we demonstrated that aged *CXCR5* knockout mice (*CXCR5*^−/−^, KO) develop retinal degeneration (RD) [8]. The degeneration was characterized by disrupted photoreceptors, upregulation of *TNFα*, presence of apoptotic cells in the retina, and by the loss of *ZO-1,* an indication of impaired blood–retinal barrier (BRB) function [9,10]. In humans, neuronal progenitor cells known to express *CXCR5*, migrated across the blood-brain barrier when exposed to *CXCL13*. The role of the *CXCL13/CXCR5* signaling in AMD has not been explored. AMD pathogenesis frequently starts with dysfunction of the retinal pigment epithelium (RPE), causing both early and late AMD [11]. Therefore, maintaining normal RPE function is of paramount significance to the prevention of AMD initiation and progression. Understanding the regulatory mechanisms underlying RPE cell dysfunction is critical to elucidate the pathophysiology of AMD. This knowledge is important in the search for new therapeutic interventions for this irreversible blinding disease.

Our previous studies showed that deficiency of *CXCR5* causes defects in RPE cells resulting in an AMD-like phenotype in mice [8,9]. A most recent study further showed that *CXCR5* signaling is required for RPE homeostasis, including establishing an epithelial phenotype, autophagy, and for mitochondrial function (unpublished). The challenge, however, is that the mechanisms by which deficiency of *CXCR5* leads to the described AMD-like phenotypes are not fully understood. In the present study, we performed a genome-wide transcriptomic analysis of WT versus *CXCR5* KO RPE cells to identify genes and pathways that mediate RPE dysfunction leading to the development of AMD. The genes and pathways identified and presented here may be important for the pathophysiology of AMD, by regulating the homeostasis of the RPE cell layer.

## 2. Results

### 2.1. Abnormalities of the CXCR5-Deficient RPE in Primary Cell Cultures

**Figure 1:**
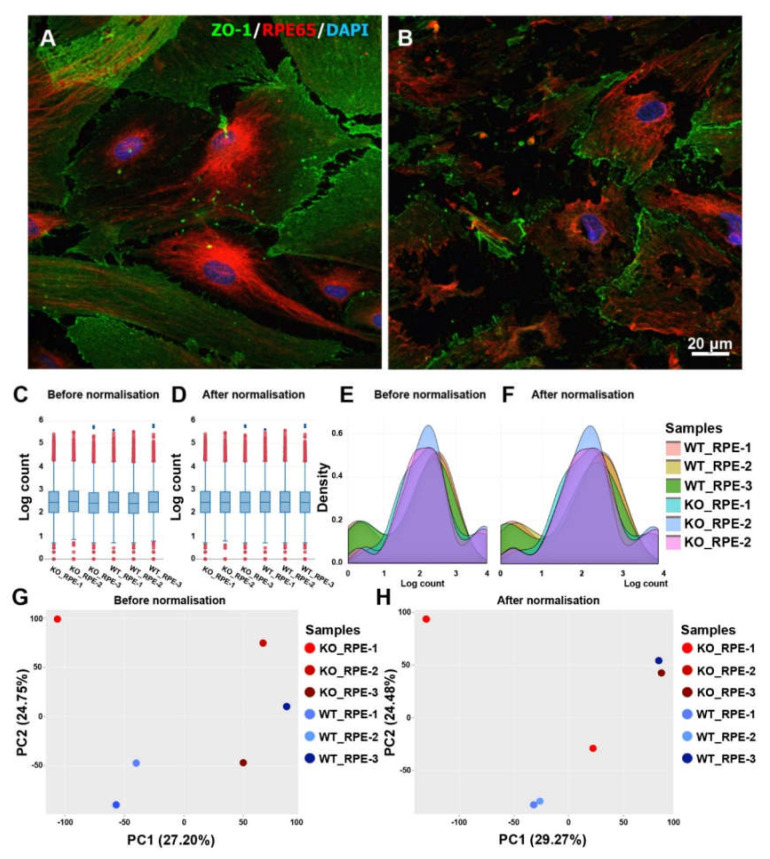
Representative double immunofluorescent staining images of *ZO-1* and RPE65 in wild-type (WT) (**A**) and *CXCR5*-deficient (**B**) retinal pigment epithelium (RPE) cells cultured on supporting cellulose substrate. Identification and correction of batch effects: (**C**) Box plot before and (**D**) after normalization; (**E**) Density plot before and (**F**) after normalization; PCA plot before (**G**) and (**H**) after normalization.

Previous studies found that deficiency of *CXCR5* leads to abnormalities in the RPE and with the presence of AMD-like phenotypes in mice [5,8,9]. The findings suggest that *CXCR5* plays a protective role in RPE cells. To test this hypothesis, here we cultured primary RPE cells derived from *CXCR5* KO mice and from C57BL6 WT controls. As shown in Figure 1A, WT RPE cells differentiated characterized, by increased expression and organization of *RPE65*, were of larger cell size, and strongly expressed *ZO-1* at cell-to-cell interaction. In addition, the cells formed a monolayer on a supporting cellulose substrate. On the contrary, RPE cells harvested and cultured from *CXCR5*^-/-^ mice did not produce a competent cell monolayer, they had poor intracellular connections, had an abnormal cell shape, and with disrupted *RPE65* organization (Figure 1B). Taken together, these *in vitro* findings are consistent with the previous *in vivo* results, and they indicate that *CXCR5* is required for RPE survival and/or homeostasis.

### 2.2. Differentially Expressed Genes in the CXCR5 KO

To explore the molecular mechanism by which *CXCR5* regulates RPE function, we performed whole transcriptome analysis in order to profile the difference between *CXCR5* KO RPE cells and WT control cells at the transcriptome level. We used a trimmed mean of M value (TMM) normalization method to count data by using DESeq2 R-package. TMM normalization adjusts the size of libraries based on the assumption that most genes are not differentially expressed. Therefore, it is essential not to make subsets of the count data before doing statistical analysis or visualization, as this can lead to differences being normalized away. Log CPM (Counts per Million) values are calculated for each gene. The CPM calculation uses the effective library size as calculated by the TMM normalization. After this initial normalization, a second normalization is then performed across samples for each gene: the counts for each gene are mean-centered and scaled to unit variance. Genes with zero expression across all samples or invalid values (NaN) are excluded. After normalization, the number of counts, density of each sample, principle component analysis (PCA), and log expression values (all2all scatter) were visualized based on the top N variable genes as defined by the coefficient of variance. Sample similarities and variance were viewed by plotting all pairwise scatter plots (Figure S1). Heatmap was used to visualize count data, and this is ideal for identifying global patterns in the data (Figure S2).

The results of count (Figure 1C,D), density plot (Figure 1E,F), PCA (Figure 1G,H), and log expression plots (Figure S1) revealed that the *CXCR5* KO RPE samples varied less compared to WT RPE. Pearson’s correlation coefficient analysis (Figure S1) and heatmap (Figure S2) indicated an overall high level of reproducibility between the three biological replicates for each sample, and within experimental groups. DESeq2 was used to assess the data to identify batch effects, and for filtering data for low count features and for removing batch effects and for performing differential analysis.

**Figure 2:**
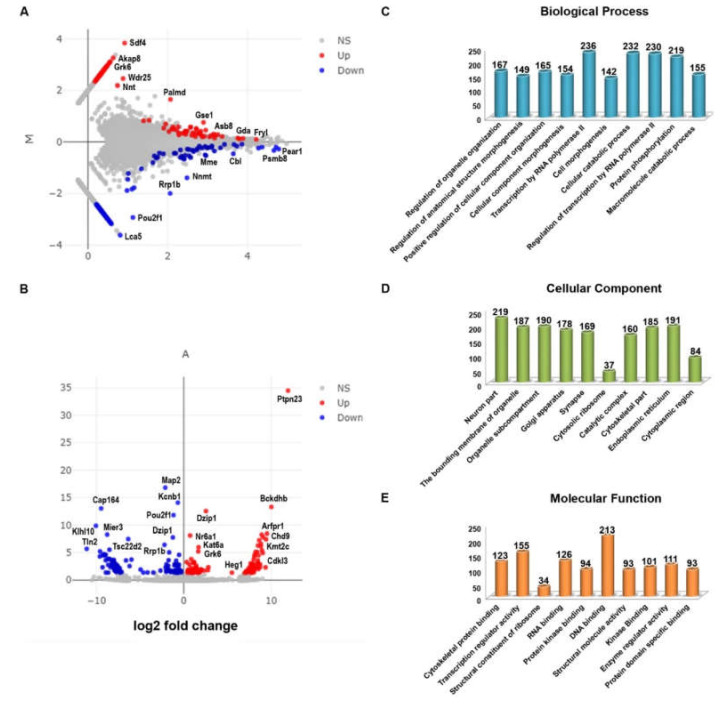
Differentially expressed genes (DEGs) visualization and gene ontology. All detected genes are plotted as an (**A**) MA plot and (**B**) volcano plot. Genes that pass a threshold of *p*-value <  0.05, padj <  0.05, and log^2^foldChange  >  0.2 in differential expression analysis were designated by red (up-regulated) and blue (down-regulated) in KO relative to WT control RPE cells. DEGs involved in various gene ontology enrichment (**C**) biological process, (**D**), cellular component, and (**E)** molecular functions, respectively. The FDR < 0.05, *p*-value < 0.05 are considered as statistical significances.

A total of 1392 differentially expressed genes (693 upregulated and 699 down-regulated genes) satisfied the criteria of FDR (False Discovery Rate) value < 1.0, fold change greater than 0.2 and less than −0.2 (logFC ± 0.2), and *p*-value less than 0.05 in *CXCR5* KO compared to WT RPE samples. A hierarchical cluster heatmap (Figure S3), MA plot (Figure 2A), and volcano plots (Figure 2B) illustrate the differentially expressed genes (DEGs). The identified DEGs were further used for gene ontology and functional pathway enrichment analysis.

### 2.3. Gene Ontology of the Differentially Expressed Genes

To further understand the functions of these DEGs, gene ontology (GO) analysis was performed. In addition, pathway enrichment analysis was performed. The identified DEGs were used as input into the DAVID tool (Database for annotation, visualization, and integrated discovery), and using the complete mouse genome as the background. Several molecular functions (MFs), biological processes (BPs), cellular components (CCs), and pathways were identified. All the GO and pathways enriched using the DEGs are presented in supplementary file 2.

GO analysis revealed that the regulation of organelle organization (GO:0033043), regulation of anatomical structure morphogenesis (GO:0022603), positive regulation of cellular component organization (GO:0051130), cellular component morphogenesis (GO:0032989), transcription by RNA polymerase II (GO:0006366), cell morphogenesis (GO:0000902), cellular catabolic process (GO:0044248), regulation of transcription by RNA polymerase II (GO:0006357), protein phosphorylation (GO:0006468), and macromolecule catabolic process (GO:0009057) were the significantly enriched GO term in biological process. The result reveals that the transcription by RNA polymerase II (236 genes), cellular catabolic process (232 genes), regulation of transcription by RNA polymerase II (230 genes), and protein phosphorylation (219 genes) were the most significantly enriched GO term in molecular functions (Figure 2C).

GO analysis for the DEGs found that the neuron part (GO:0097458), bounding membrane of organelle (GO:0098588), organelle subcompartment (GO:0031984), Golgi apparatus (GO:0005794), synapse (GO:0045202), cytosolic ribosome (GO:0022626), catalytic complex (GO:1902494), cytoskeletal part (GO:0044430), endoplasmic reticulum (GO:0005783), and cytoplasmic region (GO:0099568) were the significantly enriched GO term in cellular components. The results revealed that the neuron part (219 genes), endoplasmic reticulum (191 genes), organelle subcompartment (190 genes), bounding membrane of organelle (187 genes), and cytoskeletal part (185 genes) were the most significantly enriched GO term in cellular components (Figure 2D).

GO analysis for the DEGs found that the cytoskeletal protein binding (GO:0008092), transcription regulator activity (GO:0140110), structural constituent of ribosome (GO:0003735), RNA binding (GO:0003723), protein kinase binding (GO:0019901), DNA binding (GO:0003677), structural molecule activity (GO:0005198), kinase binding (GO:0019900), enzyme regulator activity (GO:0030234), and protein domain specific binding (GO:0019904) were significantly enriched GO term in molecular functions (Figure 2E). The result revealed that DNA binding (213 genes), transcription regulator activity (155 genes), and RNA binding (126 genes) were the most significantly enriched GO term in molecular functions (Figure 2E).

The GO results described that transcription by RNA polymerase II, catabolic process, protein phosphorylation, neuron part, endoplasmic reticulum, organelle subcompartment, cytoskeletal part of genes was more affected in the *CXCR5*-deficient RPE cells.

### 2.4. Pathway Enrichment Analysis

**Figure 3:**
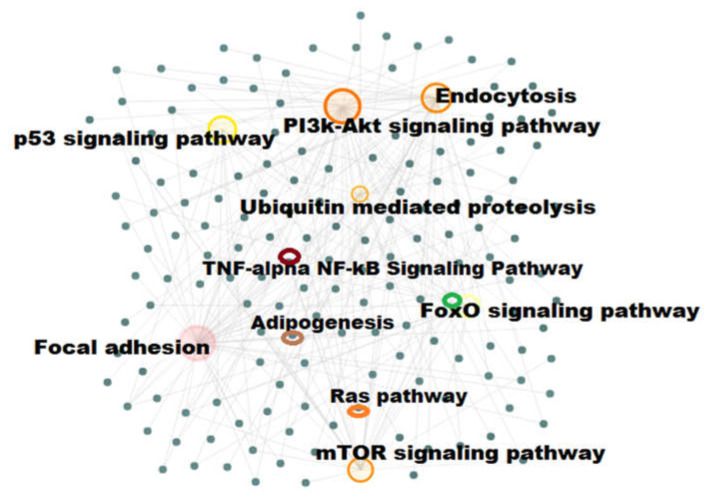
Functional pathway enrichment network analysis. The differentially expressed genes (DEGs) involved various pathways like *PI3K-Akt* signaling pathway, focal adhesion, Ras pathway, endocytosis, *p53* signaling, Adipogenesis pathway, *mTOR* signaling pathway, *FoxO* signaling pathway, ubiquitin-mediated proteolysis, *TNF-alpha NF-kB* signaling pathway respectively. The FDR < 0.05, *p*-value < 0.05 are considered as statistical significances.

**Figure 4:**
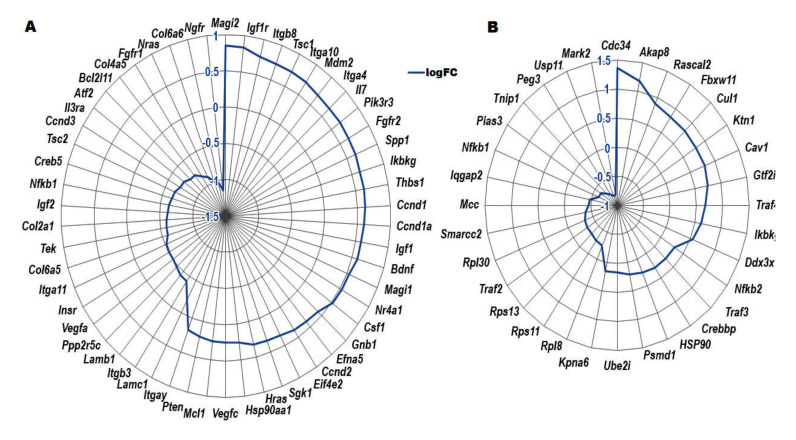
The DEGs are involved pathways like (**A**) *PI3K-Akt* signaling pathway, (**B**) *TNF-α NF-κB* signaling pathway respectively. The up-regulated and down-regulated genes extend edge to the center point were based on the logFC values. The negative values indicate the down-regulated genes, and positive values indicate the up-regulated genes in KO RPE versus WT RPE.0.

Using the identified DEGs, several pathways were enriched, including *PI3K-Akt* signaling pathway (54 genes), *FoxO* signaling pathway (19 genes), *mTOR* signaling pathway (29 genes), focal adhesion (40 genes), endocytosis (43 genes), ubiquitin-mediated proteolysis (26 genes), *TNF-alpha NF-kB* signaling pathway (32 genes), adipogenesis genes (23 genes), *p53* signaling (14 genes), and Ras Pathway (10 genes) respectively (Figure 3). From the analysis, most of DEGs were found to be involved in *PI3K-Akt* signaling pathway (Table 1), focal adhesion (Table S2), endocytosis (Table S3), *mTOR* signaling pathway (Table S4), *FoxO* signaling pathway (Table S5), *TNF-alpha,* and *NF-kB* signaling pathways (Table 2). The *PI3K-Akt* signaling pathway and *TNF-alpha NF-kB* signaling pathway genes and their logFC values are shown in Figure 4. In addition, some of the genes were found to be involved in autophagy, epithelial–mesenchymal transition (EMT), and mitochondrial pathways (Figure S4).

### 2.5. Gene–Gene Network with Pathway Enrichment Analysis

**Figure 5:**
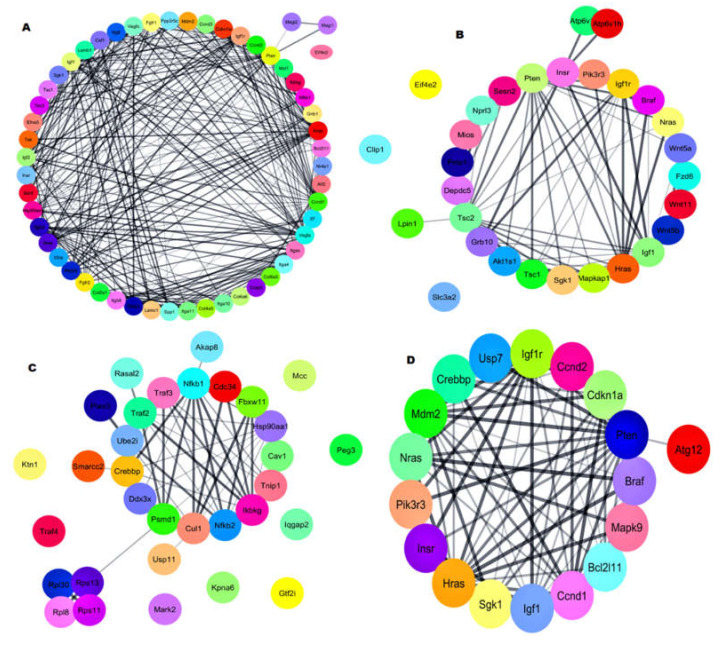
Gene–gene interaction network analysis. (**A**) *PI3K-Akt* signaling pathway genes network, (**B**) *mTOR* signaling pathway genes network, (**C**) *TNF-alpha NF-κB* Signaling Pathway genes network, and (**D**) *FoxO* signaling pathway genes network.

The DEGs were analyzed for gene–gene interaction networks using the STRING tool https://string-db.org/ (accessed on 31 May 2020) with the Cytoscape v.3.7 tool https://cytoscape.org/ (accessed on 31 May 2020). The gene–gene network for DEGs was generated with their respective minimum required interaction score (0.400). For the genes involved in the *PI3K-Akt* signaling pathway, the gene–gene network (Figure 5A) showed that 51 genes interacted directly with each other. The results revealed that 53 genes were directly involved, and one gene was indirectly involved in the *PI3K-Akt* signaling pathway. For genes in the *mTOR* signaling, pathway 26 genes were connected directly with each other, and three genes were singletons (Figure 5B). These results indicate that 26 genes were involved indirectly, and three genes were indirectly involved in the *mTOR* signaling pathway. For the genes in the focal adhesion network, 40 genes interacted directly with the main network and were involved in the Focal adhesion pathway (Figure S5A).

For the endocytosis pathway network, 33 genes were connected directly, two genes formed a subnetwork and connected to the main network, and six genes were singletons (Figure S5B). The results revealed that 35 genes were directly involved, and eight genes were involved indirectly in the endocytosis signaling pathway. The ubiquitin-mediated proteolysis network showed 25 genes were connected directly to each other, but one gene was a singleton and connected to the main network (Figure S6A). The result reveals that 26 genes were involved in the ubiquitin-mediated proteolysis pathway. The *TNF-alpha NF-kB* signaling network showed 20 genes were internally connected, four genes were involved in the subnetwork, and connected to the main network along with eight singletons (Figure 5C). The result reveals that 24 genes were directly involved, and eight genes were indirectly involved in *TNF-alpha NF-kB* Signaling Pathway. The Adipogenesis network showed one main network and three subnetworks along with six singletons (Figure S6B). The result reveals that fifteen genes were directly involved; eight genes were indirectly involved in the adipogenesis pathway. The *p53* signaling network showed 13 genes were interconnected in the main network along with one singleton (Figure S7A). The result reveals that 13 genes were directly involved, and one gene was indirectly involved in the *p53* signaling pathway. The Ras Pathway network showed only one main network, interconnected with each other in the main network without singleton (Figure S7B). The result reveals that ten genes were directly involved in the Ras signaling pathway. The *FoxO* pathway network showed eighteen genes were interconnected with each other in the main network (Figure 5D). It reveals that eighteen genes were directly involved in *FoxO* signaling pathway.

### 2.6. RT-PCR Validation of the RNA Seq Results

**Figure 6:**
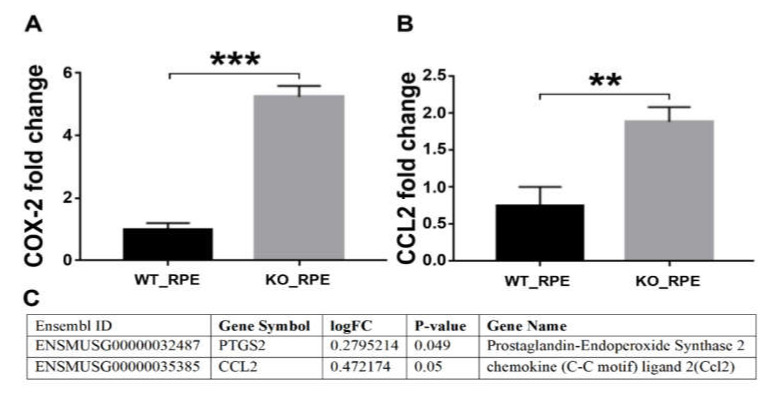
Quantitative RT-PCR analysis of (**A**) *Prostaglandin-Endoperoxide Synthase 2* (*COX-2*) and (**B**) *chemokine ligand 2* (*CCL2*) in WT and *CXCR5*-deficient RPE (n = 3). The Student’s t-test was used to determine statistical significance between the two groups. ** *p* < 0.05; *** *p* < 0.01. (**C**) RNA-seq values of logFC and *p*-value for *COX-2* and *CCL2*. The positive logFC values indicate up-regulated genes, and negative values indicate down-regulated genes in *CXCR5*-deficient RPE cells relative to WT controls.

We performed RT-PCR for the *Prostaglandin-Endoperoxide Synthase 2* (*COX-2*; Figure 6A) and *chemokine ligand 2* (*CCL2*; Figure 6B) using the same RNA that was used in sequencing to validate the quality of the RNA-seq results. The data obtained by RT-PCR were consistent with RNA seq logFC change values (Figure 6C), confirming the high quality of the transcriptome data and its analysis.

## 3. Discussion

The RPE cells play a crucial role in the survival and function of the neural retina by maintaining ionic homeostasis at the subretinal space, prevent access of blood components to the neural retina and autoimmune response, which are in part attributed to the formation of the outer blood–retina barrier (BRB) [12]. Our previous studies suggested that *CXCR5* signaling plays a protective role in the integrity of RPE cells to prevent the development of AMD-like pathological phenotypes in mice [5,8,9]. Further evidence supports the notion that *CXCR5* is required for the homeostasis of RPE cells (Figure 1A, B, and unpublished data). It is established that chemokines are chemotactic cytokines that control migration and localization of various cell types such as immune cells and leukocytes by binding to the corresponding chemokine receptors [13]. However, the mechanisms through which chemokine receptor *CXCR5* and ligand *CXCL13* signal to regulate age-related macular degeneration have not been explored. In this study, we performed transcriptome profiling in *CXCR5* KO relative to WT RPE cells. Principal component analysis, hierarchic cluster analysis, and Pearson coefficient correlation analysis indicate high sample quality and less variability among replicates and samples within the same experimental group. Using the defined DEGs, the most enriched functional pathways were: *PI3K-Akt* signaling pathway, *FoxO* signaling pathway, *mTOR* signaling pathway, focal adhesion, endocytosis, ubiquitin-mediated proteolysis, *TNF-alpha NF-kB* signaling pathway, adipogenesis genes, *p53* signaling, Ras pathway, autophagy, epithelial–mesenchymal transition (EMT), and mitochondrial pathways. These pathways were highly enriched in *CXCR5*-KO RPE and may be involved in RPE cell dysfunction.

A closer analysis of the genes involved in the *phosphatidylinositol 3-kinase (PI3K)/Akt/mTOR* pathway indicated an upregulation of genes such as *thrombospondin-1 (Thbs1)*, *G protein-coupled receptor kinase 6 (Grk6)*, *phosphoinositide-3-kinase regulatory subunit 3 (Pik3r3)*, *mitogen-activated protein kinase-associated protein 1 (Mapkap1)*, *folliculin interacting protein 1 (Fnip1), and frizzled class receptor 6 (Fzd6)*. This finding is in agreement with the study by Parrales et al. [12], which indicated that *PI3K/Akt/mTOR* signaling pathway mediates RPE cell dysfunctions. Another finding is the abnormal *FoxO1* activity in *CXCR5* KO RPE cells, as indicated by the upregulation of *Tuberous sclerosis 1 (Tsc1)* and downregulation of *Tsc2* transcripts in KO RPE cells relative to WT control cells. Cao et al. established the interaction of *FoxO1* with *Tsc2*, with the degradation of the latter, leading to decreased phosphorylation of *Akt* and *FoxO1* and insulin resistance through activation of the *mTOR* pathway [14]. Indirectly, this theory is supported by dramatically reduced Insulin receptor transcripts and increased transcription of *Insulin-like growth factor 1 (IGF-1)* in *CXCR5*-deficient RPE cells suggestive of insulin resistance. Zheng and Quirion, 2009 [15] reported that *IGF-1*, whereas binding to its receptor induces autophosphorylation, then stimulates its intrinsic tyrosine kinase, leading to phosphorylation of numerous intracellular substrates like adaptor protein *Shc* and *insulin receptor substrate-1*, followed by the initiation of several signaling pathways, such as the *phosphatidylinositol 3-kinase (PI3K)/protein kinase B (Akt)* pathway and mitogen-activated protein kinase/extracellular signal-regulated kinase (*MAPK/ERK*).

The Arf family comprises six members of Arf1–6 and Arf-like proteins. Arfs are involved in the reorganization of the actin cytoskeleton and regulation of vesicle budding. *ADP-ribosylation factor 6 (Arf6)* is a small-GTPase protein that controls the membrane trafficking between the endosome and plasma membrane [16]. *Arf1* is localized in the endosome and the Golgi, and it controls the coat protein used for intra-Golgi transport, membrane association of COPI, and retrograde transport from the cis-Golgi to the endoplasmic reticulum (ER). It also controls the membrane association of adaptor Proteins *AP-1* and *AP-3*, which are adaptors for *clathrin*. These are used for endosome-to-lysosome transport and trans-Golgi network (TGN)-to-endosome transport, respectively. However, the *Arf6* gene localizes to both the recycling endosome and the plasma membrane; then, it controls clathrin-dependent and independent endocytosis [17].

Our results identified many endocytosis genes and Golgi-related genes such as *ArfGAP* with GTPase domain, *ankyrin repeat and PH domain 3 (Agap3)*, *adaptor-related protein complex 2, mu 1 subunit (Ap2m1)*, ArfGAP with RhoGAP domain, *ankyrin repeat and PH domain 2 (Arap2)*, *ADP-ribosylation factor GTPase activating protein 1 (Arfgap1)*, *ADP-ribosylation factor GTPase activating protein 3 (Arfgap3)*, *ADP-ribosylation factor guanine nucleotide-exchange factor 2 (Arfgef2)*, *Golgi-specific brefeldin A-resistance factor 1 (Gbf1)*, *programmed cell death 6 interacting protein (Pdcd6ip)*, *vacuolar protein sorting 37A (Vps37a)*, *vacuolar protein sorting 37C (Vps37c)* and *BCL2-like 11* (apoptosis facilitator) respectively. This result correlates with Palacios et al., 2002 [18] finding that a lack of *CXCR5* gene expression leads to a deficiency in the degradation of photoreceptor outer segments (POS) by RPE cells, as of the failure of POS-containing phagosomes to acidify appropriately as an effect of inhibition of the phagosome-lysosome fusion events. Reduced rates of POS degradation by RPE cells might lead to an accumulation of unprocessed material inside the cells. Wavre-Shapton et al., 2013 [19] reported that the defects in the engulfment of POS or their subsequent degradation are associated with inherited retinal degenerative diseases. Interestingly, a recent study [20] proposes a new role for endosomes, precisely early endosomes (EEs), in controlling complement-mediated inflammation. This is appropriate for the RPE as aberrant complement activity and improved inflammation, are accompanying with age-related macular degeneration and Stargardt inherited macular dystrophy.

*TNF-α* is a major regulator of RPE activation responses, including cell attachment, spreading, chemotaxis, migration, and proliferation [21]. Jin et al., 2000 [22] described the *TNF-α* stimulates cells by activating the receptors designated as *TNF-α receptor (TNFR)* type I (*p55*) and type II (*p75*). Activation of the *TNFR* leads to a cascade of events resulting in the activation of *protein kinase A*, *protein kinase C*, *mitogen-activated protein kinase (MAPK)*, and ceramide-dependent protein kinase pathways. We have previously discussed that these MAPKs are critical in the proliferation and migration response of RPE to growth factors such as *platelet-derived growth factor (PDGF)* [22]. Marchiando et al., 2010 [23] proposed that *TNF-α* was induced a focal intrajunctional concentration of occludin followed by *caveolin-1 (CAV-1)*–dependent endocytosis in the intestinal epithelial cells. *Caveolae* have been involved in transcytosis, calcium signaling, endocytosis, and several other signal transduction events. *Nuclear transcription factor (NF)-κB* is a crucial regulator of numerous genes, as well as apoptosis-related genes and multiple inflammatory cytokine genes [21]. Xiao et al., 2003 [24] elucidated that *TNF-α*–induced apoptosis is disallowed by parallel *TNF-α*–the induced making of anti-apoptotic proteins, such as a *cellular Fas-associated death domain (FADD)-like interleukin-1β-converting enzyme-like inhibitory protein (c-FLIP)* and *cellular inhibitor of apoptosis protein (c-IAP)*, a process mediated by *NF-κB*. Shinoura et al., 2010 [25] described that the *NF-κB* inhibition consequences in apoptosis in a different type of cell types that are formerly resistant to *TNF-α*–induced apoptosis.

Similarly, our results indicate that *inhibitor of kappaB kinase gamma* (*Ikbkg*), *nuclear factor of kappa light polypeptide gene enhancer in B cells 2* (*Nfkb2*), *TNF receptor-associated factor 3* (*Traf3*) and *TNF receptor-associated factor 4* (*Traf4*) were up-regulated in *CXCR5* deficient group. TNF superfamily members transcripts (M25, M26, M10B) were also highly up-regulated in KO RPE cells. Takahashi et al. reported [26] *TNF-α*-mediated EMT in RPE cells through transforming growth factor-β (TGF-β) signaling. Previously, we have also reported increased TNFα signaling in RPE/choroid complexes of the aged *CXCR5* ko mice [8].

Among the most abundant transcripts identified in the KO RPE cells’, *p63* and *p53* family proteins (*Trp63*, *Trp53inp2*, *Trp53inp1*) that are known to be strongly associated with EMT in human keratinocytes [27], through enhanced *TGF-β* expression [28]. EMT can be suppressed by autophagy activation, thereby promoting RPE homeostasis. [29]. Autophagy is a cellular catabolic process in which damaged organelles and protein aggregates are degraded through several sequential processes: autophagy induction, autophagosome formation, autophagy-lysosome fusion, and autolysosome degradation [30]. Autophagy processes are controlled by approximately 20 transcription factors, 30 autophagy-related (*ATG*) genes, and 50 lysosomal hydrolases that are evolutionally conserved from yeast to mammal [31,32]. Autophagy processes are modulated by various pathophysiological conditions, such as amino acid starvation, and by intracellular signaling pathways, such as *PI3K/AKT/mTOR* pathway [33]. Like other cell types, RPE cells need autophagy function to maintain their homeostasis. In the RPE cells, autophagy is necessary for POS phagocytosis and visual cycle [34]. Cumulative evidence supports a protective function of autophagy for RPE cells from various stress conditions, such as oxidative damage [35]. In contrast, autophagy deregulation can cause damage to RPE cells and induce AMD pathologies, such as aberrant sub-RPE deposition and RPE degeneration [36,37].

In addition, many BRB (blood retinal barrier) transcripts were down-regulated in the *CXCR5* KO RPE cells, and they include *collagen, type II, alpha 1* (*Col2a1*), *collagen, type IV, alpha 5* (*Col4a5*), *collagen, type VI, alpha 5* (*Col6a5*), *collagen, type VI, alpha 6* (*Col6a6*), *integrin alpha 11* (*Itga11*), *integrin beta 3* (*Itgb3*), *laminin B1* (*Lamb1*) and *laminin, gamma 1* (*Lamc1*) genes (Focal adhesion pathway). In RPE cells, collagens play a crucial role in the formation of the extracellular matrix. Impairment of this matrix leads to complement pathway activation and extracellular deposition [38]. This transcriptomic data may explain the abnormal cellular shape and impaired RPE cell monolayer observed in the current study *in vitro* (Figure 1B) and the previous reports [9] of impaired blood–retinal barrier and sub-RPE deposition of AMD associated proteins in the aged *CXCR5*^-/-^ mice.

We have also observed the cytokines, chemokines, and prostaglandins such as *IL13RA1*, *IL7*, *IL13RA2*, *CCL28*, *CCL2*, *PTGDR*, *PTGES3*, *PTGER2*, *PTGIS* and *PTGS2* (*COX-2*) to be up-regulated in *CXCR5*-deficient RPE which may suggest the involvement of RPE cells in pro-inflammation signaling and immune cells recruitment. Our previous *ex-vivo* experiments in aged *CXCR5*-deficient mice have indicated increased microglia migration to RPE cell layer sub-RPE infiltration with CD4^+^ cells, as well as increased expression of *COX-2* in the mouse RPE-choroid complexes [39]. However, the exact cellular source of these genes has remained unclear. Thus, we have selected pro-inflammatory *COX-2* and chemoattractant *CCL2* genes for the transcriptome data validation. The RT-PCR results correlated with our transcriptome data analysis. In our results, up-regulation of *COX-2* in primary *CXCR5* KO RPE cultures suggestive of cellular distress, further promoting the notion of *CXCR5* to play a homeostatic role in RPE cells and consistent with our findings, such as disrupted cellular monolayer (Figure 1B). *COX-2* is a prominent inflammatory mediator and survival factor for RPE cells associated with inflammatory signaling, oxidative stress, and the promotion of autophagy [40]. The RT-PCR results have also revealed RPE cells as a direct source of *COX-2* and *CCL2* in *CXCR5*-deficient RPE cells. Studies have shown that several CC chemokines increase significantly after inflammation-associated retinal degeneration. In particular, the chemokine *CCL2* is the most well characterized. Feng et al., 2010 [41] disclosed the expression of *CCL2* and its receptor in activation and migration of microglia and monocytes induced by photoreceptor apoptosis. These findings indicate that the *CCL2* system is required in retinal degeneration associated with *CXCR5*-deficiency, with RPE cells being a significant source of *CCL2*.

In summary, our investigation reveals that deficiency of *CXCR5* abrogates *CXCL13/CXCR5* signaling and this alters the normal *PI3K-Akt* signaling pathway, *FoxO* signaling pathway, *mTOR* signaling pathway, focal adhesion, endocytosis, ubiquitin-mediated proteolysis, *TNF-alpha NF-kB* signaling pathway, adipogenesis genes, *p53* signaling, Ras pathway, autophagy, epithelial–mesenchymal transition (EMT), and mitochondrial pathways, leading to dysregulated RPE functioning, which may result in retinal degeneration that is observed in vivo [5,8,9]. The complex interplay between dysfunctional RPE, microglia, and immune cells, along with impaired blood–retinal barrier, requires further investigations. Still, existing data is strongly suggestive of a positive inflammatory feedback loop. Where lack of *CXCR5* induce RPE homeostatic dysregulation, leading to increased pro-inflammatory signaling and photoreceptor apoptosis. These changes promote the recruitment of microglia and immune cells that can gain access to sub-RPE space and retina through an incompetent RPE monolayer. Immune cell activation produces more inflammatory and cell death, thus closing the loop.

## 4. Materials and Methods

### 4.1. Animals

All experiments with mice were approved by the Institutional Animal Care and Use Committee (IACUC #9520) of the University of Missouri at 9 April 2019, and all animal experiments were conducted according to the guidelines of the ARVO (Association for Research in Vision and Ophthalmology). The (C57BL/6J) (WT) and (B6.129S2(Cg)-*CXCR5*tm1Lipp/J) (*CXCR5*-/-, KO) https://www.jax.org/strain/006659 (accessed on 31 May 2020) mice strains were purchased from Jackson Laboratory. We maintained all mice in specific pathogen-free animal facilities at the Bond Life Sciences Center, University of Missouri, USA, with fed normal chow diets and provided with water *ad libitum*. Genotyping was outsourced to TransnetYX (Cordova, TN, USA) www.transnetyx.com (accessed on 31 May 2020). Animals were validated for *CXCR5* knockout and for the presence of the neomycin resistance gene. All mice were screened for the presence of Rd8-associated nucleotide deletion in the *Crumbs homolog 1 (CRB1)* gene, as reported previously [9] and found to be Rd8 mutation-negative. The correctness of resulting datasets was also validated by checking for the presence of *CXCR5* transcripts in all wild-type control and absence in *CXCR5* knockout datasets (Table 3).

### 4.2. Primary RPE Cell Cultures and Treatment

Following mice sacrifice by CO_2_ inhalation, RPE cells were isolated from adult (6-mo-old) WT and KO mice. This was done by carefully dissecting the eye globe on ice to remove the anterior chamber and retina. The resulting eyecup was then incubated with trypsin Gibco™ Trypsin-EDTA (0.25%) for 40 min, with the RPE cell layer facing down. Following initial digestion, RPE cells were released by gentle shaking of the eyecup using sterile forceps. The isolated RPE cells were then cultured with an N1 complete medium in a 6-well plate coated with Attachment Factor™ (4Z0-201; Cell Systems). Cells were grown according to the protocol described by Johnson et al., for primary human RPE [42]. Cells were grown until robust pigmented RPE monolayer was formed.

### 4.3. Immunofluorescence Staining

For immunohistochemistry evaluation, the isolated RPE cells were grown on cellulose matrix with a pore size of 0.45 µm (R9NA09917, Millicell, MilliporeSigma, Burlington, MA, USA) for four weeks in N1 media. The protocol of growing RPE cell culture on the porous substrate was a modification of a previous protocol described by Johnson et al., described for primary human RPE [43]. The substrate is meant to imitate Bruch’s membrane. After four weeks of growth, the cells were fixed with HistoChoice Molecular Biology fixative (VWR Life Science, Missouri, TX, USA) for 10 min, permeabilized with 0.05% Triton X-100 for 10 min, and blocked with 5% normal goat serum (NGA; 50062Z, Thermo Fisher Scientific, Waltham, MA, USA) for 1 hour at RT. The samples were then incubated with the primary antibody, RPE65 (1:50; MA1-16578; Thermo Fisher Scientific, Waltham, MA), and *ZO-1* (1:100; 61-7300; Thermo Fisher Scientific, Waltham, MA, USA). The samples were then washed with Phosphate-Buffer-Saline (PBS; 1967729, Thermo Fisher Scientific, Waltham, MA, USA) and 0.05% Tween-20 (PBS-T; 1392C184; Amresco, Solon, OH, USA) and then incubated with secondary antibody: goat anti-rabbit IgG (H + L), Cyanine5 (A10523, 1:1000; Thermo Fisher Scientific, Waltham, MA, USA), goat anti-rabbit IgG (H+L), Alexa Fluor 488 (A-11034, 1:1000, Thermo Fisher Scientific, Waltham, MA, USA) and goat anti-mouse IgG (H + L), Cyanine5 (A10524, 1:1000, Thermo Fisher Scientific, Waltham, MA, USA). The cell nuclei were stained counterstained with 4′,6-diamidino-2-phenylindole (DAPI; 1:5000; D9542 MilliporeSigma, Burlington, MA, USA). ProLong Diamond antifade reagent (Thermo Fisher Scientific, Waltham, MA, USA) was used for mounting.

### 4.4. Imaging

Fluorescent images were acquired using a LeicaSP8 laser confocal microscope (Leica AG, Wetzlar, Germany). Max intensity projection was used in representative figures.

### 4.5. RNA Isolation and Sequencing

Confluent RPE cells were harvested for the total RNA isolation using RNA Easy Mini RNA Isolation Kit (Qiagen, Germantown, MD, USA). Isolated RNA was submitted to the Novogene Leading Edge Genomic Services & Solutions, (Sacramento, CA, USA) for library preparation and sequencing. In summary, Agilent 2100 Bioanalyzer was used to verify the quality of the isolated RNA. DNAse treatment was performed to eliminate DNA from the sample. The Illumina HiSeq 2500 was used for RNA sequencing. The sequencing experiment was performed according to the manufacturer’s instructions (Illumina, San Diego, CA, USA). TruSeq library generation kits were used. Library construction was performed using random fragmentation of the poly A^+^ mRNA, along with cDNA production using random primers. Clusters were produced nearly 725K–825K clusters/mm^2^. The run after the first base addition parameters were assessed, then cluster density and quality determined. The cDNA sequences were aligned to the reference genome mouse (mm10), and then paired-end sequencing runs were performed. A total of approximately 15 million paired, 50 bp reads per sample were obtained. The raw RNA-Seq datasets are deposited to the NCBI-SRA (Sequence Read Archive (SRA) with accession numbers, SRA No: SRP251872; BioProject: PRJNA610827.

### 4.6. Bioinformatics Analysis

A total of six RNA-seq datasets was generated, three from *CXCR5* knockout (KO RPE_1, KO RPE _2, KO RPE _3), and three from control (WT RPE_1, WT RPE _2, WT RPE _3) RPE cells. The raw fastq reads were trimmed for adapters and pre-processed to remove low-quality reads using Trimmomatic v.0.36 using the default parameter setting for Illumina paired-end reads. Cleaning and trimming of low-quality reads and adaptor removal lead to retention of more than 96% of good quality reads in each stage (Table S1).

The raw data was used for visualization of reads before and after pre-processing by using FastQC software: https://www.bioinformatics.babraham.ac.uk/projects/fastqc/ (accessed on 31 May 2020). The distribution of the raw sequences was quantified. The processed datasets were mapped to the mouse reference genome (mm10) with HISAT2 http://daehwankimlab.github.io/hisat2/ (accessed on 31 May 2020). with default parameters [44]. The alignment results showed that 98.15% reads aligned with the reference genome.

### 4.7. Gene Expression Analysis

Gene expression was calculated using high-quality reads. Differentially expressed genes were identified using the DESeq2 v 1.20 package in R software v 3.2.2 http://www.R-project.org (accessed on 31 May 2020). Both WT and KO comparison transcript counts (matrix file) were used for differential gene expression analysis using the DESeq2 package of Bioconductor with primary parameters such as FDR (false discovery rate), logFC (log fold-change) and *p*-value set at default. Unigenes with an FDR (False Discovery Rate) value < 1.0, fold change greater than 0.2 and less than −0.2 (logFC ± 0.2), and the *p*-value is less than 0.05 were considered as significantly differentially expressed genes (DEGs). These genes were used for further functional gene annotation analysis.

### 4.8. Functional Annotation

Gene ontology (GO) enrichment analysis was initially performed using the DAVID annotation database (The Database for Annotation, Visualization, and Integrated Discovery, https://david.ncifcrf.gov/ (accessed on 31 May 2020) [45,46]. Functional Annotation Clustering was used for the molecular function (MF), biological process (BP), and cellular component (CC) categorization [47,48]. GO terms that had the lowest *p*-values in each cluster, as well as low FDR values, were selected for further analysis. The total number of genes included in all of the listed GO terms was considered as significantly enriched within the gene set.

Protein-protein network analysis using the DEGs was performed using the STRING 10.5 database https://string-db.org/ (accessed on 31 May 2020) outputs such as Gene Ontology, KEGG, Pfam, and InterPro were analyzed. STRING software employs a Fisher’s exact test, followed by a correction for multiple testing [49,50].

### 4.9. Quantitative RT-PCR

The same RNA samples described in 4.5 above were reverse transcribed to cDNA using SuperScript VILO cDNA Synthesis Kit (Invitrogen, Thermo Fisher Scientific, Waltham, MA, USA) in SimpliAmp Thermal Cycler (Thermo Fisher Scientific, Waltham, MA). SYBR green chemistry was used with SYBR Green Master Mix (4344463, Thermo Fisher Scientific, Waltham, MA, USA). The following mouse-specific primers were used to amplify target genes: *COX-2* by Sanchez et al., [51] (forward: GCGAGCTAAGAGCTTCAGGA, reverse: CAGACGCCACTGTCGCTTT), *CCL2* by Schiller et al., [52] (forward: GAAGGAATGGGTCCAGACAT, reverse: ACGGGTCAACTTCACATTCA), and *GAPDH* as "housekeeping" gene by Tsujita et al., [53] (forward: CATGGCCTTCCGTGTTCCTA, reverse: TGTCATCATACTTGGCAGGTTTC) in a Quant Studio 3 RT-PCR system (Applied Biosystems, Thermo Fisher Scientific, Waltham, MA, USA). The expression of the target genes was normalized to that of *GAPDH*. Fold change was calculated using the relative quantification (2−ΔΔCT) method. Three biological replicates per group were used with three technical replicates for each experiment.

### 4.10. Statistical Analysis

All numeric values were expressed as the mean ± standard deviation (SD) for the respective groups. Statistical analysis was performed using the DESeq2 R-package https://bioconductor.org/packages/release/bioc/html/DESeq2.html (accessed on 31 May 2020). The Students’ test was used when comparing two groups. Benjamin–Hochberg corrections were also used to correct for multiple comparisons. A *p*-value of less than 0.05 was considered significant.

## 5. Statement of Limitations

1. The small number of samples in this study limits the depth of the identified DEGs.
2. Significant variability of the obtained samples, as indicated by Fig. 1G, H. The cell culture system is sensitive to various factors such as environmental factors during sample isolation, culture, and extraction. The primary cell culture has also inherited variability from the animals that have become the donors of the cells. This aspect further limits the dept of this study.
3. Freshly isolated cells may be a preferable source of RNA to study the effects of *CXCR5* in the RPE. However, fresh isolates of mouse RPE are technically challenging to produce due to the low number of cells available from each eye. The pooling of the significant number of mice is required to achieve levels of RNA suitable for sequencing. Mechanical isolation produces isolates of the poor purity with contamination from choroidal vasculature and other cell types. Trypsin dissociation has an issue of purity in the immediate dissociate (culture in N1 media produce positive proliferative pressure promoting growth and differentiation specifically of RPE cells but no other cell types, i.e., endothelial cells). Trypsin exposure produces its effects on various genes and pathways (i.e., activation of *Protease-activated receptor 2* pathway). Primary RPE cultures could exclude indirect or paracrine effects from other retinal tissues (i.e., choriocapillaris, microglia, and photoreceptors) and rule out the systemic effect of global *CXCR5* knockout.

## 6. Conclusions

In conclusion, a transcriptome-wide gene expression signature of mouse RPE cells is described based on extensive bioinformatics analysis of RNA sequencing data. The study reveals a list of candidate genes that may be important for mediating retinal/macular diseases. These genes may represent potential molecular markers for assessing the integrity and function of RPE. These findings give insight into the molecular signaling mechanisms of *CXCR5* deficiency in RPE cell dysfunction and in the pathophysiology of AMD disease.

## Figures and Tables

**Table 1:** List of *PI3K-Akt* signaling pathway genes with logFC, *p*-value, and FDR value.

Gene Symbol	Gene Name	logFC ^1^	*p*-value	FDR
Atf2	Activating transcription factor 2	−0.76676	0.000148	0.041635
Bcl2l11	BCL2-like 11 (apoptosis facilitator)	−0.76676	0.000148	0.041635
Bdnf	Brain derived neurotrophic factor	0.579128	0.002926	0.27166
Ccnd1	Cyclin D1	0.619593	0.016261	0.72475
Ccnd2	Cyclin D2	0.381798	0.033778	0.986212
Ccnd3	Cyclin D3	−0.70571	0.014237	0.672932
Cdkn1a	Cyclin-dependent kinase inhibitor 1A (P21)	0.600435	0.000799	0.129915
Col2a1	Collagen, type II, alpha 1	−0.60353	0.012355	0.627982
Col4a5	Collagen, type IV, alpha 5	−0.85519	0.001471	0.187435
Col6a5	Collagen, type VI, alpha 5	−0.58554	0.022181	0.835681
Col6a6	Collagen, type VI, alpha 6	−1.03427	0.000611	0.108132
Creb5	cAMP responsive element binding protein 5	−0.64907	0.030466	0.947107
Csf1	Colony-stimulating factor 1 (macrophage)	0.515929	0.024112	0.860187
Efna5	Ephrin A5	0.402745	0.015585	0.709264
Eif4e2	Eukaryotic translation initiation factor 4E member 2	0.339331	0.017923	0.756945
Fgfr1	Fibroblast growth factor receptor 1	−0.9058	0.0000018	0.001257
Fgfr2	Fibroblast growth factor receptor 2	0.641456	0.033897	0.986212
Gnb1	Guanine nucleotide binding protein (G protein), beta 1	0.424488	0.006647	0.453973
Hras	Harvey rat sarcoma virus oncogene	0.319113	0.041055	0.999738
Hsp90aa1	Heat shock protein 90, alpha (cytosolic), class A member 1	0.256992	0.042641	0.999738
Igf1	Insulin-like growth factor 1	0.582664	0.011964	0.618509
Igf1r	Insulin-like growth factor I receptor	0.839659	0.001664	0.199427
Igf2	Insulin-like growth factor 2	−0.6181	0.035299	0.999738
Ikbkg	Inhibitor of kappaB kinase gamma	0.624192	0.00867	0.521805
Il3ra	Interleukin 3 receptor, alpha chain	−0.70596	0.016436	0.727482
Il7	Interleukin 7	0.65812	0.030148	0.946044
Insr	Insulin receptor	−0.50178	0.0276	0.914738
Itga10	Integrin, alpha 10	0.722545	0.005989	0.424382
Itga11	Integrin alpha 11	−0.52376	0.040259	0.999738
Itga4	Integrin alpha 4	0.669187	0.021709	0.828055
Itgav	Integrin alpha V	0.17317	0.02295	0.84715
Itgb3	Integrin beta 3	−0.42099	0.032828	0.976771
Itgb8	Integrin beta 8	0.758588	0.001698	0.200273
Lamb1	Laminin B1	−0.43077	0.000041	0.015966
Lamc1	Laminin, gamma 1	−0.19129	0.031532	0.958818
Magi1	Membrane-associated guanylate kinase, WW and PDZ domain containing 1	0.534198	0.030475	0.947107
Magi2	Membrane-associated guanylate kinase, WW and PDZ domain containing 2	0.852149	0.004337	0.355457
Mcl1	Myeloid cell leukemia sequence 1	0.239522	0.000743	0.123992
Mdm2	Transformed mouse 3T3 cell double minute 2	0.707868	0.002289	0.234591
Nfkb1	Nuclear factor of kappa light polypeptide gene enhancer in B cells 1, p105	−0.6222	0.000512	0.096041
Ngfr	Nerve growth factor receptor (TNFR superfamily, member 16)	−1.14937	0.00000013	0.000158
Nr4a1	Nuclear receptor subfamily 4, group A, member 1	0.533577	0.034039	0.988161
Nras	Neuroblastoma ras oncogene	-0.98599	0.000281	0.064468
Pik3r3	Phosphoinositide-3-kinase regulatory subunit 3	0.655702	0.005241	0.393197
Ppp2r5c	Protein phosphatase 2, regulatory subunit B’, gamma	−0.4741	0.013059	0.642713
Pten	Phosphatase and tensin homolog	0.207488	0.029398	0.933135
Sgk1	Serum/glucocorticoid regulated kinase 1	0.331703	3.24 × 10^−5^	0.012944
Spp1	Secreted phosphoprotein 1	0.640272	0.003407	0.3029
Tek	TEK receptor tyrosine kinase	−0.59019	0.030625	0.949013
Thbs1	Thrombospondin 1	0.62293	0.000441	0.086938
Tsc1	Tuberous sclerosis 1	0.727053	0.003532	0.307612
Tsc2	Tuberous sclerosis 2	−0.65896	0.029703	0.937812
Vegfa	Vascular endothelial growth factor A	−0.50133	8.23 × 10^−5^	0.027091
Vegfc	Vascular endothelial growth factor C	0.251123	0.000599	0.106649

^1^ The positive values indicate up-regulated genes, and negative values indicate down-regulated genes in CXCR5-deficient RPE cells relative to WT controls.

**Table 2:** List of *TNF-alpha NF-kB* Signaling Pathway genes with logFC, *p*-value, and FDR values.

Gene Symbol	Gene Name	logFC ^1^	*p*-value	FDR
Akap8	A kinase (PRKA) anchor protein 8	1.175046	0.000030	0.012441
Cav1	Caveolin 1, caveolae protein	0.79122	0.009721	0.554537
Cdc34	Cell division cycle 34	1.35894	0.00000051	0.000446
Crebbp	CREB binding protein	0.289525	0.015087	0.698369
Cul1	Cullin 1	0.815439	0.007716	0.49339
Ddx3x	DEAD/H (Asp-Glu-Ala-Asp/His) box Polypeptide 3, X-linked	0.54556	0.005442	0.401934
Fbxw11	F-box and WD-40 domain protein 11	0.824636	0.00000084	0.000016
Gtf2i	General transcription factor II I	0.752596	0.002818	0.265882
Hsp90aa1	Heat shock protein 90, alpha (cytosolic), class A member 1	0.256992	0.042641	0.999738
Ikbkg	Inhibitor of kappaB kinase gamma	0.624192	0.00867	0.521805
Iqgap2	IQ motif containing GTPase activating protein 2	−0.4761	0.007342	0.478612
Kpna6	Karyopherin (importin) alpha 6	0.160461	0.04708	0.999738
Ktn1	kinectin 1	0.793005	0.001688	0.199531
Mark2	MAP/microtubule affinity regulating kinase 2	−0.82436	0.001453	0.186526
Mcc	Mutated in colorectal cancers	-0.45668	0.003974	0.334461
Nfkb1	Nuclear factor of kappa light polypeptide gene enhancer in B cells 1, p105	−0.6222	0.000512	0.096041
Nfkb2	Nuclear factor of kappa light polypeptide gene enhancer in B cells 2, p49/p100	0.305921	0.015427	0.708003
Peg3	Paternally expressed 3	−0.76858	0.001785	0.206978
Pias3	Protein inhibitor of activated STAT 3	−0.63029	0.035361	0.999738
Psmd1	Proteasome (prosome, macropain) 26S subunit, non-ATPase, 1	0.213848	0.002695	0.258467
Rasal2	RAS protein activator like 2	0.89088	0.000071	0.024327
Rpl30	Ribosomal protein L30	−0.34108	0.000105	0.032262
Rpl8	Ribosomal protein L8	−0.25082	0.027523	0.912855
Rps11	Ribosomal protein S11	−0.2783	0.023677	0.856335
Rps13	Ribosomal protein S13	−0.31715	0.000249	0.059475
Smarcc2	SWI/SNF-related, matrix associated, actin-dependent regulator of chromatin, subfamily c, member 2	−0.39488	0.008876	0.52407
Tnip1	TNFAIP3 interacting protein 1	−0.71759	0.018253	0.762899
Traf2	TNF receptor-associated factor 2	−0.31858	0.027154	0.906937
Traf3	TNF receptor-associated factor 3	0.302541	0.025658	0.886335
Traf4	TNF receptor associated factor 4	0.663643	0.02293	0.84715
Ube2i	Ubiquitin-conjugating enzyme E2I	0.161077	0.021286	0.815485
Usp11	Ubiquitin specific peptidase 11	−0.82071	0.000582	0.105223

^1^ The positive values indicate upregulated genes, and negative values indicate down-regulated genes in CXCR5-deficient RPE cells relative to WT controls.

**Table 3:** Number of *CXCR5* transcripts in WT and *CXCR5* KO datasets.

Dataset	WT_RPE-1	RPE_WT-1	RPE_WT-1	KO_RPE-1	KO_RPE-2	KO_RPE-3
ENSMUSG00000047880 (*CXCR5*) Number of transcripts per 10^6^ reads	109	341	316	0	0	0

## Supplementary Materials

Supplementary materials can be found at https://www.mdpi.com/2227-9059/8/6/147/s1.
