# Supplementary material for: Transcriptome-Wide Analysis of CXCR5 Deficient Retinal Pigment Epithelial (RPE) Cells Reveals Molecular Signatures of RPE Homeostasis
Source: Biomedicines. 2020 Jun 1;8(6):147. doi: 10.3390/biomedicines8060147 (PMC7345337; doi:10.3390/biomedicines8060147)
Supplement: Supplementary file 1 [file biomedicines-08-00147-s001.pdf]

biomedicines

Article

Transcriptome-wide analysis of CXCR5 deficient retinal pigment epithelial (RPE) cells reveals molecular signature of RPE homeostasis

Supplementary data

Madhu Sudhana Saddala [1], Anton Lennikov [1], Anthony Mukwaya [2], and Hu Huang [1,*]

1 Department of Ophthalmology, University of Missouri, Columbia, MO 65212, United States of America; saddalam@missouri.edu (M.S.S.); lennikova@missouri.edu (A.L.)

2 Department of Ophthalmology, Institute for Clinical and Experimental Medicine, Faculty of Health Sciences, Linköping University, Linköping, 58183 , Sweden; anthonny.mukwaya@liu.se

* Correspondence:huangh1@missouri.edu; Tel: +1 573-882-9899

# Madhu Sudhana Saddala and Anton Lennikov have contributed equally to this work.

Keywords: Age-related macular degeneration; CXCR5; EMT, FoxO; Mitochondria; RNA-Seq, Gene Ontology; KEGG; Retinal pigment epithelium

Supplementary Figures

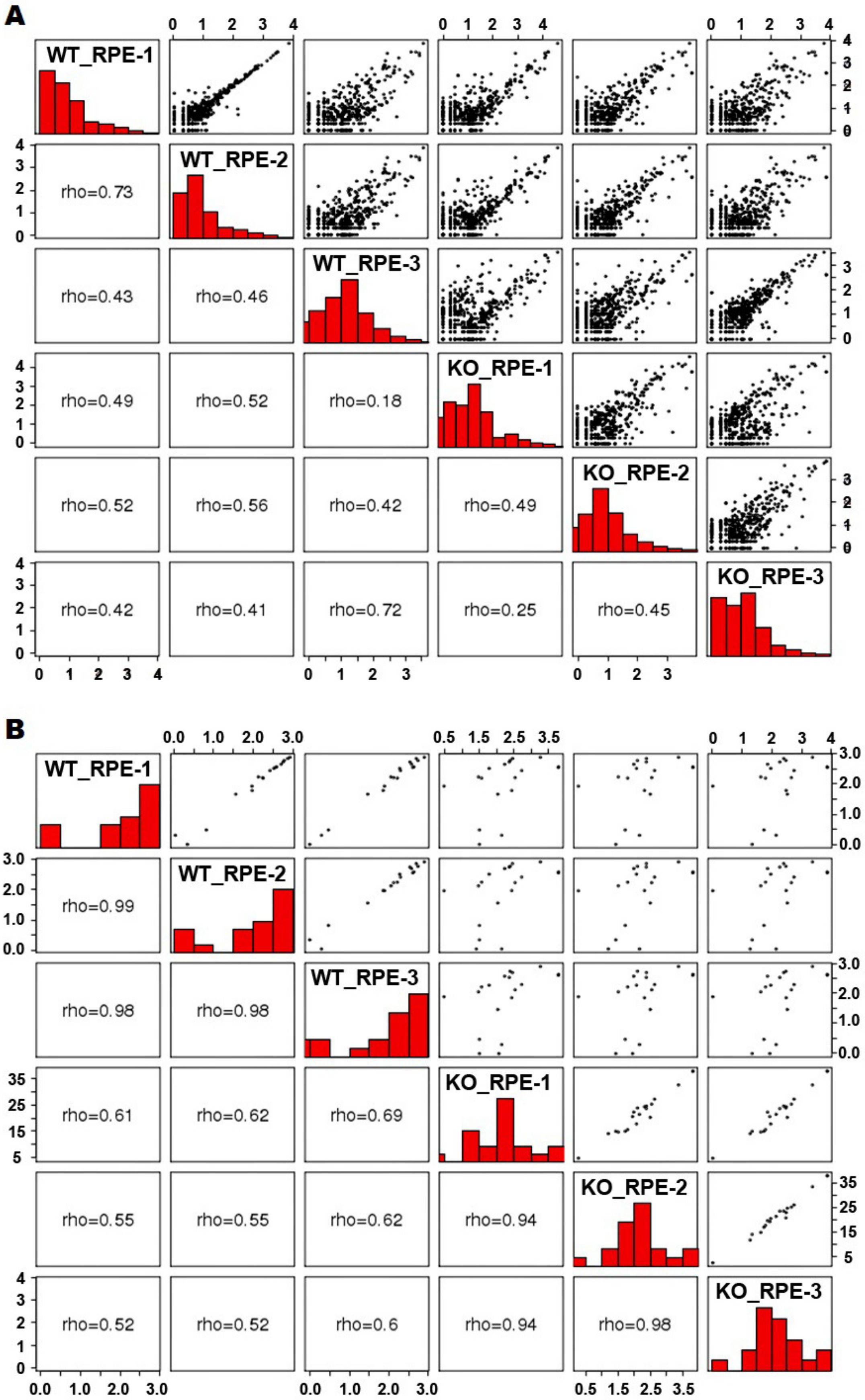
A
WT_RPE-1
WT_RPE-2
WT_RPE-3
KO_RPE-1
KO_RPE-2
KO_RPE-3
rho=0.73
rho=0.43
rho=0.46
rho=0.49
rho=0.52
rho=0.18
rho=0.52
rho=0.56
rho=0.42
rho=0.49
rho=0.42
rho=0.41
rho=0.72
rho=0.25
rho=0.45
B
WT_RPE-1
WT_RPE-2
WT_RPE-3
KO_RPE-1
KO_RPE-2
KO_RPE-3
rho=0.99
rho=0.98
rho=0.98
rho=0.61
rho=0.62
rho=0.69
rho=0.55
rho=0.55
rho=0.62
rho=0.94
rho=0.52
rho=0.52
rho=0.6
rho=0.94
rho=0.98

Figure S1: Quantitative profiling of mouse RPE tissue between control and CXCR5 ko groups. Pearson correlation coefficient plot of the log2 ratio between two groups (A) before and (B) after normalization. Potential relationships or correlations amongst the different data attributes is to leverage a pair-wise correlation matrix in control and CXCR5 knockout groups.

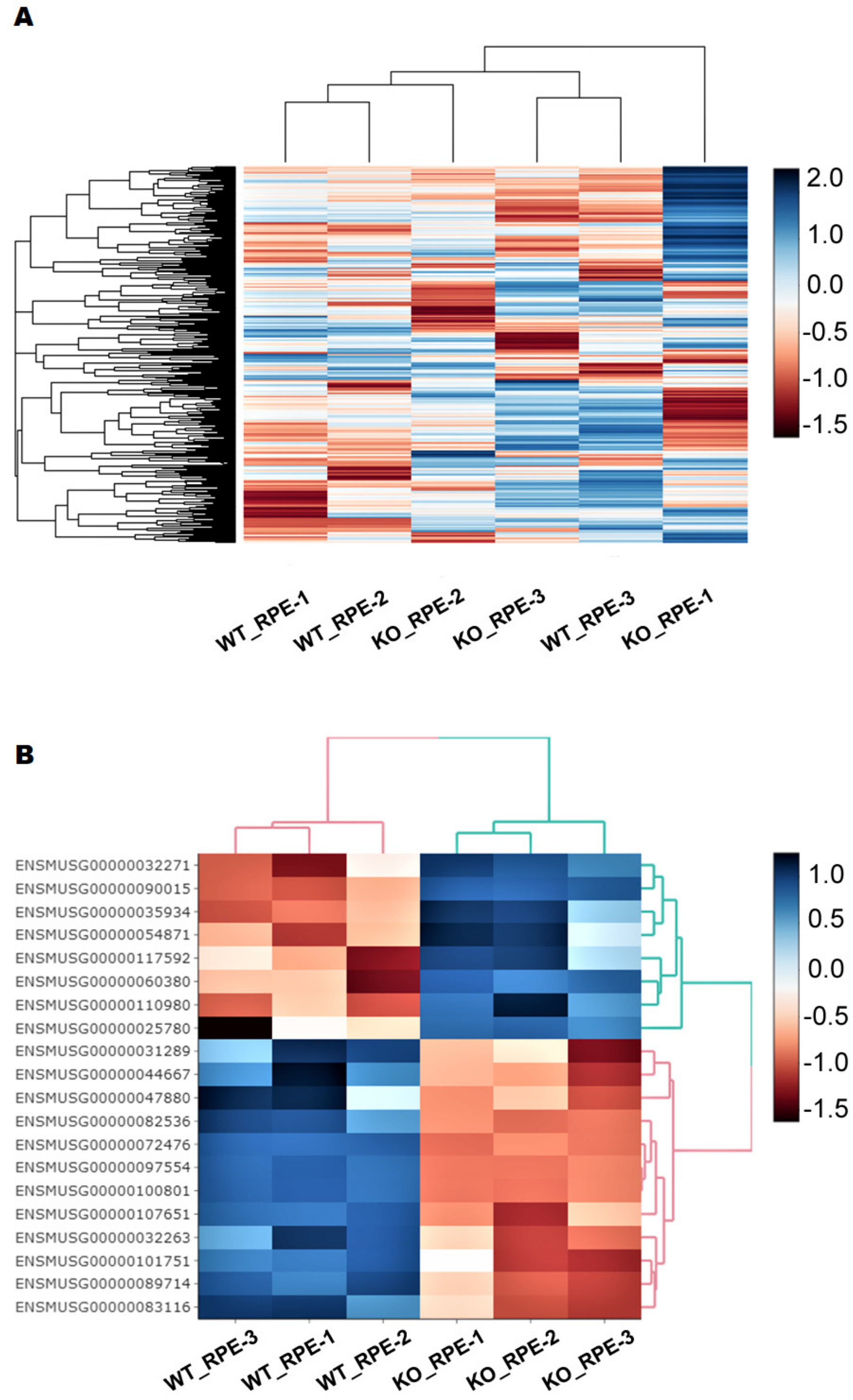
A
2.0
1.0
0.0
-0.5
-1.0
-1.5
WT_RPE-1
WT_RPE-2
KO_RPE-2
KO_RPE-3
WT_RPE-3
KO_RPE-1
B
ENSMUSG00000032271
ENSMUSG00000090015
ENSMUSG00000035934
ENSMUSG00000054871
ENSMUSG00000117592
ENSMUSG00000060380
ENSMUSG00000110980
ENSMUSG00000025780
ENSMUSG00000031289
ENSMUSG00000044667
ENSMUSG00000047880
ENSMUSG00000082536
ENSMUSG00000072476
ENSMUSG00000097554
ENSMUSG00000100801
ENSMUSG00000107651
ENSMUSG00000032263
ENSMUSG00000101751
ENSMUSG00000089714
ENSMUSG00000083116
1.0
0.5
0.0
-0.5
-1.0
-1.5
WT_RPE-3
WT_RPE-1
WT_RPE-2
KO_RPE-1
KO_RPE-2
KO_RPE-3

Figure S2: Potential relationships or correlations amongst the different data attributes is to leverage a pair-wise correlation matrix in control and CXCR5 knockout groups. (A) heatmap of total differentially expressed genes (B) The cluster of main heatmap represented twenty differentially expressed genes. The color code for the scored data is shown above the heat map. The red color denoted with a down-regulated genes and blue color denoted with up-regulated genes.

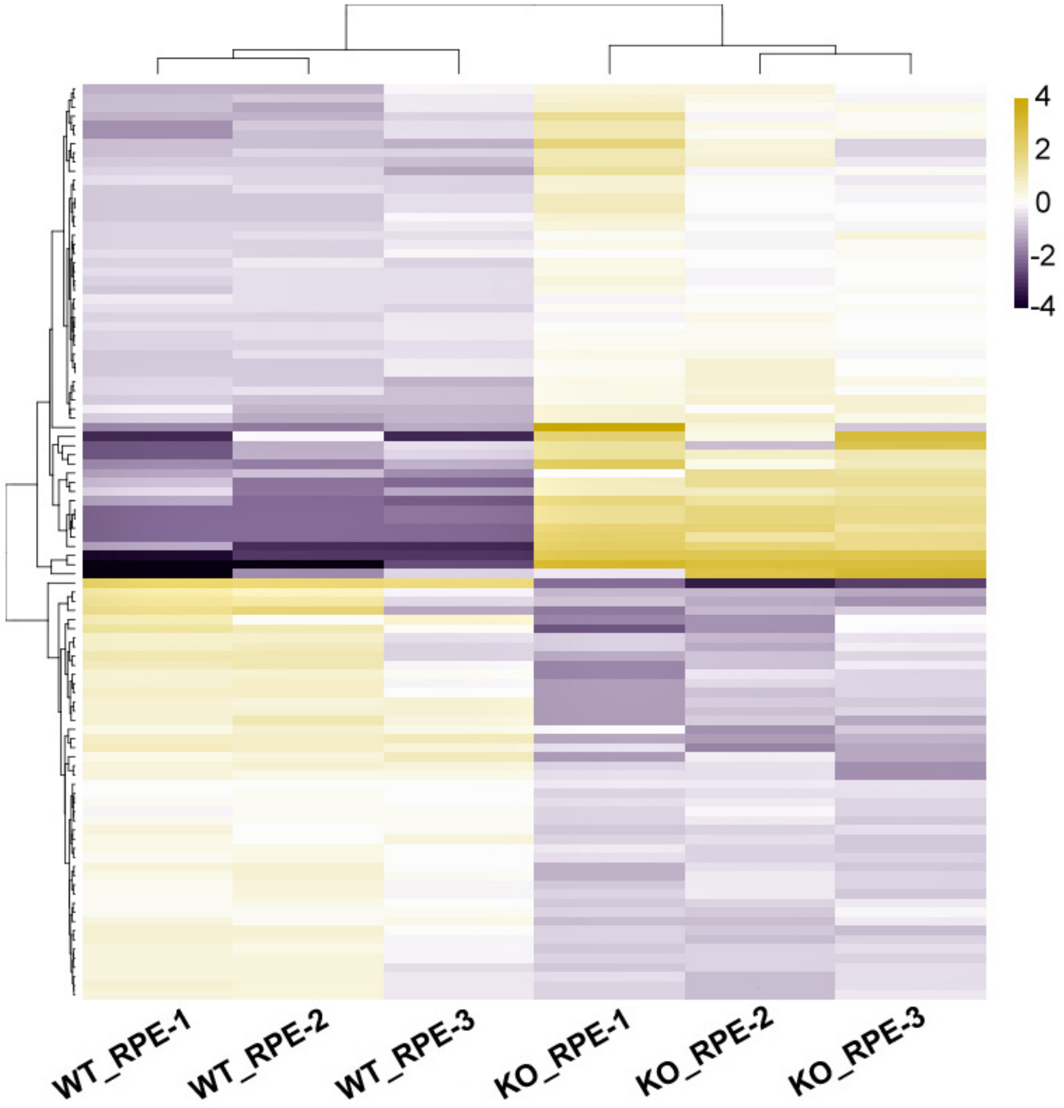

Figure S3: Significantly hundred differentially expressed genes were represented as a heat map (cluster of Suppl. Figure 2A), yellow color shows up-regulated and purple color shows down-regulated genes.

A

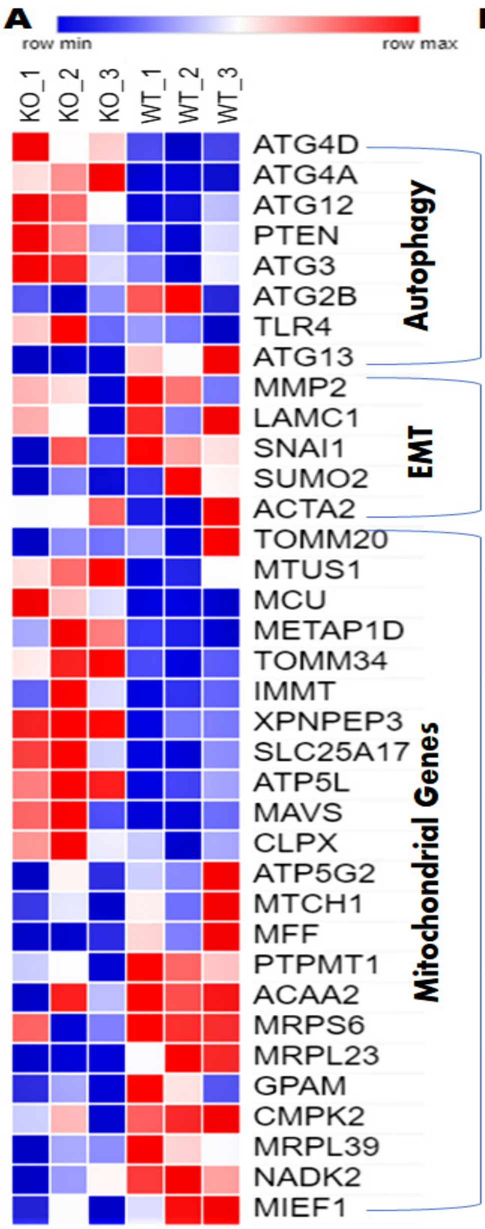

B

Gene	LogFC	P-value	FDR
**Autophagy**			
ATG4D	0.683624	0.004788	0.371858
ATG4A	0.487587	0.001627	0.196834
ATG12	0.313796	0.00212	0.225683
TLR4	0.312007	0.001452	0.03194
PTEN	0.207488	0.029398	0.933135
ATG3	0.202362	0.035314	0.999738
ATG2B	0.182891	0.079267	0.971331
ATG13	-0.50177	0.022987	0.84715
**EMT**			
MMP2	-0.34852	3.92E-07	2.58E-05
LAMC1	-0.20566	0.000735	0.024255
SNAI1	-0.30021	0.02391	0.151882
SUMO2	-0.38004	0.01058	0.11638
ACTA2	0.284318	0.03608	0.170091
**Mitochondrial genes**			
TOMM20	-0.1632	0.004743	0.062613
MTUS1	0.644678	0.032367	0.970279
MCU	0.462395	0.031205	0.956413
METAP1D	0.445701	0.035845	0.999738
TOMM34	0.423444	0.02489	0.874241
IMMT	0.407843	0.042487	0.999738
XPNPEP3	0.398445	0.003731	0.320286
SLC25A17	0.35216	0.000157	0.043256
ATP5L	0.316717	0.002646	0.255228
MAVS	0.266238	0.025026	0.877256
CLPX	0.216659	0.020204	0.797743
ATP5G2	-0.21962	0.037978	0.999738
MTCH1	-0.27155	0.000958	0.143022
MFF	-0.27277	0.032176	0.968435
PTPMT1	-0.30452	0.010882	0.587004
ACAA2	-0.34139	0.022319	0.837896
MRPS6	-0.3878	0.025537	0.885838
MRPL23	-0.4002	0.048774	0.999738
GPAM	-0.54439	0.008822	0.523758
CMPK2	-0.61277	0.00432	0.355457
MRPL39	-0.63066	0.033787	0.986212
NADK2	-0.67435	0.020821	0.807156
MIEF1	-0.78074	0.010224	0.565986

Figure S4: The DEGs involved in (**A**) autophagy, Epithelial-Mesenchymal Transition (EMT) and mitochondrial pathways (heatmap), (**B**) genes, logFC, p-value and FDR values. The heatmap designates red color as up-regulated and blue color as down-regulated genes in CXCR5-deficient RPE cells relative to WT control.

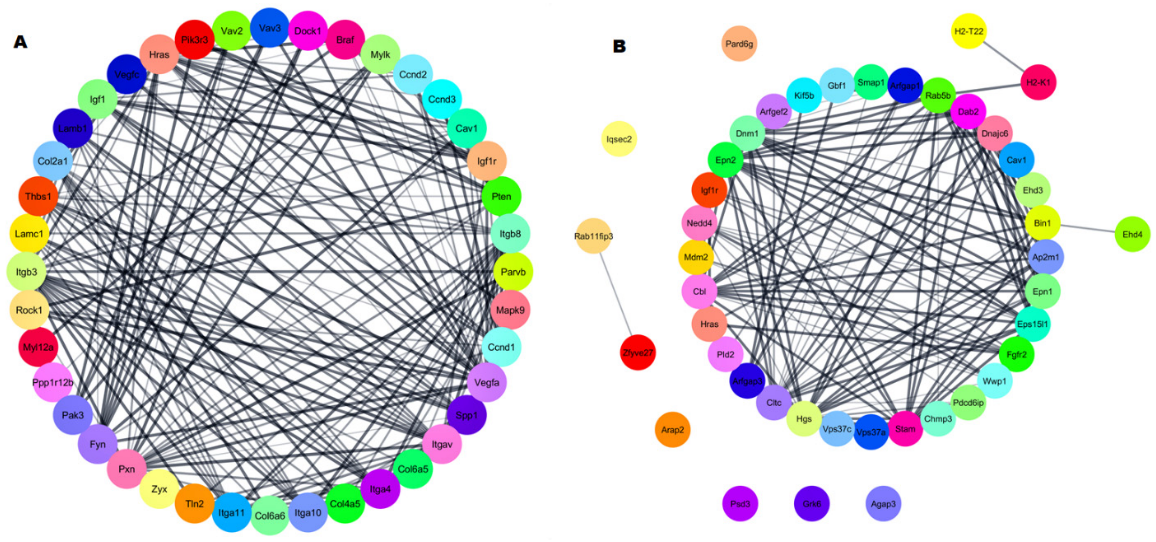

Figure S5: Gene-gene interaction network analysis. **(A)** Focal adhesion pathway genes. **(B)** Endocytosis pathway genes.

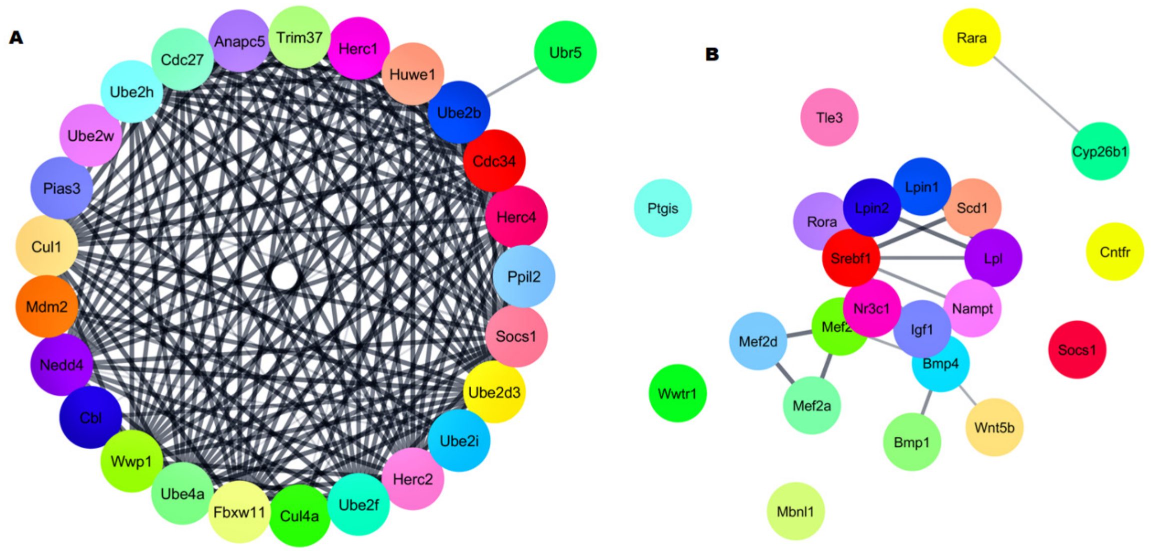

Figure S6: Gene-gene interaction network analysis. (**A**) Ubiquitin pathway genes. (**B**) Adipogenesis pathway genes.

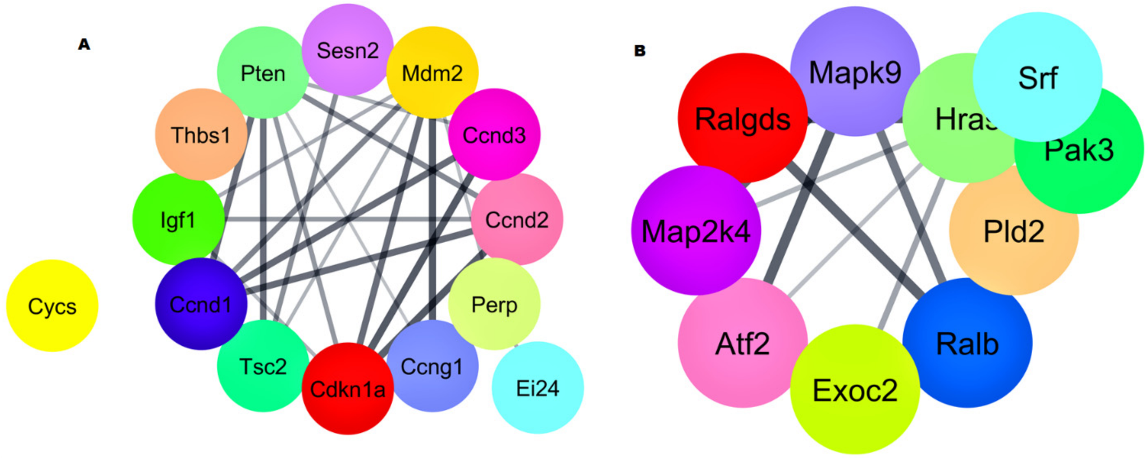

Figure S7: Gene-gene interaction network analysis. (**A**) p53 pathway genes. (**B**) Ras pathway genes.

Supplementary Tables

Table S1: Trimmomatic for trimming Illumina FASTQ data and removing sequence adapters.

Samples	Trimmomatic trimming reads		Drop of reads	Survival percentage of reads (%)
	Before	After		
RPEWT_1_L1_1	29276152	28197164	1078988	96.32
RPEWT_1_L1_2	29276152	28197164	1078988	96.32
RPEWT_2_L2_1	26792021	25764597	1027424	96.17
RPEWT_2_L2_2	26792021	25764597	1027424	96.17
RPEWT_3_L2_1	29958156	28923170	1034986	96.55
RPEWT_3_L2_2	29958156	28923170	1034986	96.55
RPECXCR5_1_L2_1	30697606	29617540	1080066	96.49
RPECXCR5_1_L2_2	30697606	29617540	1080066	96.49
RPECXCR5_2_L2_1	31530713	30295204	1235509	96.08
RPECXCR5_2_L2_2	31530713	30295204	1235509	96.08
RPECXCR5_3_L2_1	27742490	26649285	1093205	96.06
RPECXCR5_3_L2_2	27742490	26649285	1093205	96.06

Table S2: List of Focal adhesion pathway genes with logFC, p-value and FDR value.

Gene Symbol	Gene Name	logFC	P-value	FDR
Braf	Braf transforming gene	0.52977	0.028286	0.924117
Cav1	Caveolin 1, caveolae protein	0.79122	0.009721	0.554537
Ccnd1	Cyclin D1	0.819543	0.001353	0.178882
Ccnd2	Cyclin D2	0.550356	0.003991	0.334988
Ccnd3	Cyclin D3	-0.70571	0.014237	0.672932
Col2a1	Collagen, type II, alpha 1	-0.60353	0.012355	0.627982
Col4a5	Collagen, type IV, alpha 5	-0.85519	0.001471	0.187435
Col6a5	Collagen, type VI, alpha 5	-0.58554	0.022181	0.835681
Col6a6	Collagen, type VI, alpha 6	-1.03427	0.000611	0.108132
Dock1	Dedicator of cytokinesis 1	-0.72792	0.00884	0.523758
Fyn	Fyn proto-oncogene	-0.3561	0.006077	0.427738
Hras	Harvey rat sarcoma virus oncogene	0.319113	0.041055	0.999738
Igf1	Insulin-like growth factor 1	0.582664	0.011964	0.618509
Igf1r	Insulin-like growth factor I receptor	0.839659	0.001664	0.199427
Itga10	integrin, alpha 10	0.722545	0.005989	0.424382
Itga11	Integrin alpha 11	-0.52376	0.040259	0.999738
Itga4	Integrin alpha 4	0.669187	0.021709	0.828055
Itgav	Integrin alpha V	0.17317	0.02295	0.84715
Itgb3	Integrin beta 3	-0.42099	0.032828	0.976771
Itgb8	Integrin beta 8	0.758588	0.001698	0.200273
Lamb1	Laminin B1	-0.43077	4.18E-05	0.015966
Lamc1	Laminin, gamma 1	-0.19129	0.031532	0.958818
Mapk9	Mitogen-activated protein kinase 9	-0.63709	0.037388	0.999738
Myl12a	Myosin, light chain 12A, regulatory, non-sarcomeric	0.487208	0.002373	0.238982
Mylk	Myosin, light polypeptide kinase	-2.17604	3.16E-25	5.51E-21
Pak3	p21 (RAC1) activated kinase 3	0.694405	0.000394	0.082617
Parvb	Parvin, beta	-0.59728	0.028586	0.927543
Pik3r3	Phosphoinositide-3-kinase regulatory subunit 3	0.655702	0.005241	0.393197
Ppp1r12b	Protein phosphatase 1, regulatory subunit 12B	0.990421	0.000248	0.059475
Pten	Phosphatase and tensin homolog	0.207488	0.029398	0.933135
Pxn	Paxillin	-0.48393	0.006902	0.46301
Rock1	Rho-associated coiled-coil containing protein kinase 1	0.403765	0.009553	0.548533

Spp1	Secreted phosphoprotein 1	0.640272	0.003407	0.3029
Thbs1	Thrombospondin 1	0.62293	0.000441	0.086938
Tln2	Talin 2	-5.05373	1.78E-70	9.33E-66
Vav2	Vav 2 oncogene	0.560233	0.037862	0.999738
Vav3	Vav 3 oncogene	-0.83643	0.006246	0.436735
Vegfa	Vascular endothelial growth factor A	-0.50133	8.23E-05	0.027091
Vegfc	Vascular endothelial growth factor C	0.251123	0.000599	0.106649
Zyx	Zyxin	0.331246	0.005829	0.420451

[1] The positive values indicate up-regulated genes, and negative values indicate down-regulated genes in CXCR5-deficient RPE cells relative to WT controls.

Table S3: List of Endocytosis pathway genes with logFC, p-value and FDR value.

Gene Symbol	Gene Name	logFC	P-value	FDR
Agap3	ArfGAP with GTPase domain, ankyrin repeat and PH domain 3	0.472514	0.03436	0.993067
Ap2m1	Adaptor-related protein complex 2, mu 1 subunit	0.235135	0.019347	0.788484
Arap2	ArfGAP with RhoGAP domain, ankyrin repeat and PH domain 2	0.539452	0.012804	0.637988
Arfgap1	ADP-ribosylation factor GTPase activating protein 1	0.598167	0.020056	0.796902
Arfgap3	ADP-ribosylation factor GTPase activating protein 3	-0.22345	0.042507	0.999738
Arfgef2	ADP-ribosylation factor guanine nucleotide-exchange factor 2 (brefeldin A-inhibited)	-0.70729	1.33E-05	0.006275
Bin1	Bridging integrator 1	-0.41634	0.002254	0.231417
Cav1	Caveolin 1, caveolae protein	0.79122	0.009721	0.554537
Cbl	Casitas B-lineage lymphoma	-1.19727	3.91E-08	4.99E-05
Chmp3	Charged multivesicular body protein 3	0.70947	0.018809	0.77501
Cltc	Clathrin, heavy polypeptide (Hc)	0.628175	0.031378	0.958818
Dab2	Disabled 2, mitogen-responsive phosphoprotein	0.394081	0.007187	0.473408
Dnajc6	DnaJ heat shock protein family (Hsp40) member C6	0.571836	0.013292	0.647561
Dnm1	Dynamin 1	-0.77705	0.009459	0.544977
Ehd3	EH-domain containing 3	0.601715	0.002116	0.225683
Ehd4	EH-domain containing 4	0.294066	0.025567	0.886143
Epn1	Epsin 1	-0.33443	0.049664	0.999738
Epn2	Epsin 2	0.649684	0.033892	0.986212
Eps15l1	Epidermal growth factor receptor pathway	0.84856	0.004189	0.347096

	substrate 15-like 1			
Fgfr2	Fibroblast growth factor receptor 2	0.641456	0.033897	0.986212
Gbf1	Golgi-specific brefeldin A-resistance factor 1	0.289734	0.030258	0.946044
Grk6	G protein-coupled receptor kinase 6	1.137069	5.11E-05	0.018864
H2-K1	Histocompatibility 2, K1, K region	-0.45265	0.013726	0.66025
H2-T22	Histocompatibility 2, T region locus 22	-0.38692	0.016101	0.720927
Hgs	HGF-regulated tyrosine kinase substrate	0.330257	0.002811	0.265882
Hras	Harvey rat sarcoma virus oncogene	0.319113	0.041055	0.999738
Igf1r	Insulin-like growth factor I receptor	0.839659	0.001664	0.199427
Iqsec2	IQ motif and Sec7 domain 2	-0.61004	0.047881	0.999738
Kif5b	Kinesin family member 5B	0.240581	0.005744	0.418374
Mdm2	Transformed mouse 3T3 cell double minute 2	0.707868	0.002289	0.234591
Nedd4	Neural precursor cell expressed, developmentally down-regulated 4	0.464203	0.046913	0.999738
Pard6g	Par-6 family cell polarity regulator gamma	0.318767	0.009863	0.559003
Pdcd6ip	Programmed cell death 6 interacting protein	0.612938	0.000449	0.087793
Pld2	Phospholipase D2	0.705743	0.020714	0.806734
Psd3	Pleckstrin and Sec7 domain containing 3	0.618409	0.027502	0.912732
Rab11fip3	RAB11 family interacting protein 3 (class II)	0.880577	0.000249	0.059475
Rab5b	RAB5B, member RAS oncogene family	-0.41939	0.015551	0.708775
Smap1	Small ArfGAP 1	-0.6663	0.028345	0.924519
Stam	Signal transducing adaptor molecule (SH3 domain and ITAM motif) 1	0.65634	0.023574	0.856219
Vps37a	Vacuolar protein sorting 37A	0.241828	0.016802	0.732061
Vps37c	Vacuolar protein sorting 37C	-0.68304	0.010018	0.562388
Wwp1	WW domain containing E3 ubiquitin protein ligase 1	0.488816	0.035938	0.999738
Zfyve27	Zinc finger, FYVE domain containing 27	0.212258	0.047278	0.999738

[1] The positive values indicate up-regulated genes, and negative values indicate down-regulated genes in CXCR5-deficient RPE cells relative to WT controls.

Table S4: List of mTOR signaling pathway genes with logFC, p-value, and FDR values.

Gene Symbol	Gene Name	logFC	P-value	FDR
Akt1s1	AKT1 substrate 1 (proline-rich)	-0.79272	0.000912	0.140096
Atp6v1b2	ATPase, H+ transporting, lysosomal V1 subunit B2	0.150992	0.040314	0.999738
Atp6v1h	ATPase, H+ transporting, lysosomal V1 subunit H	0.290488	0.013095	0.642713
Braf	Braf transforming gene	0.52977	0.028286	0.924117
Clip1	CAP-GLY domain containing linker protein 1	0.415577	0.023969	0.860121
Depdc5	DEP domain containing 5	-0.63296	0.007948	0.499113
Eif4e2	Eukaryotic translation initiation factor 4E member 2	0.339331	0.017923	0.756945
Fnip1	Folliculin interacting protein 1	0.600433	0.042345	0.999738
Fzd6	Frizzled class receptor 6	0.871764	0.002598	0.253124
Grb10	Growth factor receptor bound protein 10	-0.69377	0.004018	0.336169
Hras	Harvey rat sarcoma virus oncogene	0.319113	0.041055	0.999738
Igf1	Insulin-like growth factor 1	0.582664	0.011964	0.618509
Igf1r	Insulin-like growth factor I receptor	0.839659	0.001664	0.199427
Insr	Insulin receptor	-0.50178	0.0276	0.914738
Lpin1	Lipin 1	0.842541	0.002497	0.246194
Mapkap1	Mitogen-activated protein kinase associated protein 1	0.465516	0.023405	0.853275
Mios	Meiosis regulator for oocyte development	-0.75748	0.00268	0.25751
Nprl3	Nitrogen permease regulator-like 3	0.411513	0.011277	0.595943
Nras	Neuroblastoma ras oncogene	-0.98599	0.000281	0.064468
Pik3r3	Phosphoinositide-3-kinase regulatory subunit 3	0.655702	0.005241	0.393197
Pten	Phosphatase and tensin homolog	0.207488	0.029398	0.933135
Sesn2	Sestrin 2	0.211571	0.03607	0.999738
Sgk1	Serum/glucocorticoid regulated kinase 1	0.331703	3.24E-05	0.012944
Slc3a2	Solute carrier family 3 (activators of dibasic and neutral amino acid transport), member 2	0.616334	0.043362	0.999738
Tsc1	tuberous sclerosis 1	0.727053	0.003532	0.307612
Tsc2	Tuberous sclerosis 2	-0.65896	0.029703	0.937812
Wnt11	Wingless-type MMTV integration site family, member 11	-0.81981	0.001098	0.156251
Wnt5a	Wingless-type MMTV integration site family, member 5A	-0.70649	0.000767	0.126041
Wnt5b	Wingless-type MMTV integration site family, member 5B	-0.57215	0.022638	0.841847

[1] The positive values indicate up-regulated genes, and negative values indicate down-regulated genes in CXCR5-deficient RPE cells relative to WT controls.

Table S5: List of FoxO signaling pathway genes with logFC, p-value and FDR values.

Gene Symbol	Gene Name	logFC[1]	P-value	FDR
Mapk9	Mitogen-activated protein kinase 9	-0.63709	0.037388	0.999738
Pten	Phosphatase and tensin homolog	0.207488	0.029398	0.933135
Hras	Harvey rat sarcoma virus oncogene	0.319113	0.041055	0.999738
Ccnd2	Cyclin D2	0.550356	0.003991	0.334988
Braf	Braf transforming gene	0.52977	0.028286	0.924117
Igf1	Insulin-like growth factor	0.582664	0.011964	0.618509
Ccnd1	Cyclin D1	0.819543	0.001353	0.178882
Pik3r3	Phosphatidylinositol 3 kinase, regulatory subunit, polypeptide 3 (p55)	0.655702	0.005241	0.393197
Igf1r	Insulin-like growth factor I receptor	0.839659	0.001664	0.199427
Nras	Neuroblastoma ras oncogene	-0.98599	0.000281	0.064468
Cdkn1a	Cyclin-dependent kinase inhibitor 1A (P21)	0.600435	0.000799	0.129915
Mdm2	Transformed mouse 3T3 cell double minute 2	0.707868	0.002289	0.234591
Bcl2l11	BCL2-like 11 (apoptosis facilitator)	-0.85382	0.001046	0.152302
Insr	Insulin receptor	-0.50178	0.0276	0.914738
Sgk1	Serum/glucocorticoid regulated kinase 1	0.331703	3.24E-05	0.012944
Crebbp	CREB binding protein	0.289525	0.015087	0.698369
Usp7	Ubiquitin specific peptidase 7	0.50216	0.001425	0.184667
Atg12	Autophagy related 12	0.313796	0.00212	0.225683

[1] The positive values indicate up-regulated genes, and negative values indicate down-regulated genes in CXCR5-deficient RPE cells relative to WT controls.

© 2020 by the authors. Submitted for open access publication under the terms and conditions of the Creative Commons Attribution (CC BY) license (http://creativecommons.org/licenses/by/4.0/).
